# Triterpenoid Saponins from the Cultivar “Green Elf” of *Pittosporum tenuifolium*

**DOI:** 10.3390/molecules26226805

**Published:** 2021-11-11

**Authors:** David Pertuit, Anne-Claire Mitaine-Offer, Tomofumi Miyamoto, Chiaki Tanaka, Christine Belloir, Loïc Briand, Marie-Aleth Lacaille-Dubois

**Affiliations:** 1 PEPITE EA 4267, Laboratoire de Pharmacognosie, UFR des Sciences de Santé, Université de Bourgogne Franche-Comté, BP 87900, CEDEX, 21079 Dijon, France; David.Pertuit@u-bourgogne.fr (D.P.); marie-aleth.lacaille-dubois@u-bourgogne.fr (M.-A.L.-D.); 2 Graduate School of Pharmaceutical Sciences, Kyushu University, Fukuoka 812-8582, Japan; miyamoto@phar.kyushu-u.ac.jp (T.M.); ctanaka@phar.kyushu-u.ac.jp (C.T.); 3 Centre des Sciences du Goût et de l’Alimentation, AgroSup Dijon, CNRS, INRAE, Université de Bourgogne Franche-Comté, CEDEX, 21065 Dijon, France; christine.belloir@inrae.fr (C.B.); loic.briand@inrae.fr (L.B.)

**Keywords:** *Pittosporum tenuifolium*, Pittosporaceae, barringtogenol C, TAS1R2/TASR3, sweet taste, taste inhibitor

## Abstract

Four oleanane-type glycosides were isolated from a horticultural cultivar “Green Elf” of the endemic *Pittosporum tenuifolium* (Pittosporaceae) from New Zealand: three acylated barringtogenol C glycosides from the leaves, with two previously undescribed 3-*O*-β-d-glucopyranosyl-(1→2)-[α-l-arabinopyranosyl-(1→3)]-β-d-glucuronopyranosyl-21-*O*-angeloyl-28-*O*-acetylbarringtogenol C, 3-*O*-β-d-galactopyranosyl-(1→2)-[α-l-arabinopyranosyl-(1→3)]-β-d-glucuronopyranosyl-21-*O*-angeloyl-28-*O*-acetylbarringtogenol C, and the known 3-*O*-β-d-glucopyranosyl-(1→2)-[α-l-arabinopyranosyl-(1→3)]-β-d-glucuronopyranosyl-21-*O*-angeloyl-28-*O*-acetylbarringtogenol C (Eryngioside L). From the roots, the known 3-*O*-β-d-glucopyranosyl-(1→2)-β-d-galactopyranosyl-(1→2)-β-d-glucuronopyranosyloleanolic acid (Sandrosaponin X) was identified. Their structures were elucidated by spectroscopic methods including 1D- and 2D-NMR experiments and mass spectrometry (ESI-MS). According to their structural similarities with gymnemic acids, the inhibitory activities on the sweet taste TAS1R2/TAS1R3 receptor of an aqueous ethanolic extract of the leaves and roots, a crude saponin mixture, 3-*O*-β-d-glucopyranosyl-(1→2)-[α-l-arabinopyranosyl-(1→3)]-β-d-glucuronopyranosyl-21-*O*-angeloyl-28-*O*-acetylbarringtogenol C, and Eryngioside L were evaluated.

## 1. Introduction

**Figure 1:**
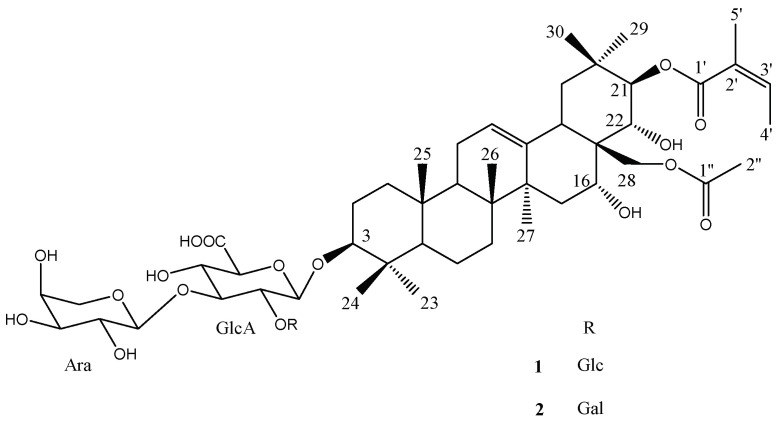
Structures of compounds **1** and **2**.

The Pittosporaceae family, belonging to the Apiales order according to APGIII classification, is distributed from tropical Africa to the Pacific islands. The genus *Pittosporum*, which comprises about 200 species, is well known for its horticultural uses. From a phytochemical point of view, these species are rich in triterpene-type glycosides with biological interests, such as antimicrobial, antioxidant, cytotoxic, and antiproliferative activities [1,2,3]. Among this species, *P. tenuifolium* Banks & Sol. ex Gaertn., native to New Zealand, is a small tree named Kohuhu in Maori. The fresh gum resin is traditionally used for its scent, and it can be mixed with thickened juice of Puha (*Sonchus* genus) and chewed as a masticatory [4]. Moreover, several cultivars of this species are sold in greenhouses for garden use. Thus, as a continuation of our study on the glycosylated derivatives from the *Pittosporum* genus [5,6,7], we investigated the phytochemical interest of the “Green Elf” cultivar of *P. tenuifolium*. In the present paper, we report the isolation and structural elucidation of two undescribed triterpene saponins (Figure 1), together with a known one from the leaves and a known one from the roots. Their structures were elucidated by spectroscopic methods, including 600 MHz 1D and 2D experiments (^1^H, ^13^C, HSQC, HMBC, COSY, TOCSY, and ROESY) in combination with mass spectrometry (ESI-MS), through a comparison of their physical and spectral data with literature values.

When we compare the structure of the isolated triterpene-type glycosides with gymnemic acids, some similarities appeared. Accordingly, we tested the inhibitory activity on the sweet taste TAS1R2/TAS1R3 receptor of an aqueous ethanolic extract of the leaves and roots, a crude saponin mixture, the pure compound **1**, and Eryngioside L.

## 2. Results and Discussion

The aqueous ethanolic extract of the leaves of *P. tenuifolium* was fractionated by vacuum liquid chromatography (VLC) and purified by several medium-pressure liquid chromatography (MLPC) runs on normal- and reverse-phase silica gel, as well as semi-preparative HPLC, yielding compounds **1**–**2** and the known Eryngioside L [8]. The known Sandrosaponin X [9] was also isolated from an aqueous ethanolic extract of the roots using the same protocol. All compounds were obtained as amorphous powders. Their structures were established mainly by spectroscopic methods including 600 MHz NMR experiments and mass spectrometry, and the structural analysis of the newly identified compounds is detailed below.

The monosaccharides were identified by extensive 2D-NMR analysis (COSY, TOCSY, ROESY, HSQC, HMBC) as glucuronopyranosyl, glucopyranosyl, and arabinopyranosyl units for **1** and as glucuronopyranosyl, galactopyranosyl, and arabinopyranosyl units for **2**. Their absolute configurations were determined to be d for glucuronic acid (GlcA), glucose (Glc), and galactose (Gal) and l for arabinose (Ara), according to their optical rotation [10] (see Section 3). The relatively large ^3^*J*_H-1,H-2_ values of GlcA, Glc, Gal, and Ara (7.0−7.6 Hz) indicated a β anomeric orientation for GlcA, Glc, and Gal and an α anomeric orientation for Ara.

Compound **1** exhibited in the HR-ESI-MS a quasi-molecular ion peak at *m*/*z* 1107.5359 [M + Na]^+^ (calcd. 1107.5352) compatible with a molecular weight of 1084 and, thus, a molecular formula of C_54_H_84_O_22_. The HSQC spectrum of the aglycone of **1** displayed seven correlations due to seven angular methyl groups at *δ*_H_/*δ*_C_ 1.19 (s)/27.6 (CH_3_-23), 1.09 (s)/16.4 (CH_3_-24), 0.82 (s)/15.4 (CH_3_-25), 0.98 (s)/16.8 (CH_3_-26), 1.81 (s)/27.1 (CH_3_-27), 1.12 (s)/29.5 (CH_3_-29), and 1.33 (s)/19.9 (CH_3_-30) (Table 1).

**Figure 2:**
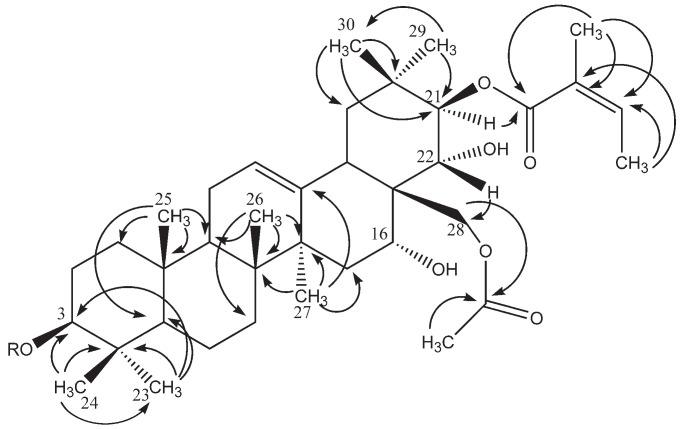
Key HMBC correlations of the aglycone moiety of compound **1**.

**Figure 3:**
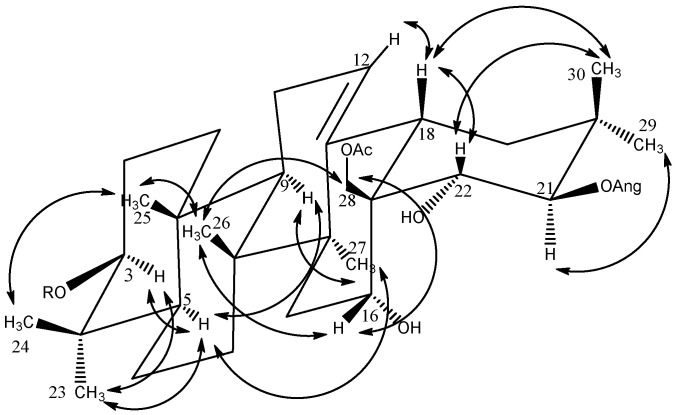
Key ROESY correlations of the aglycone moiety of compound **1**.

Furthermore, some characteristic signals were observed: one olefinic proton at *δ*_H_/*δ*_C_ 5.50 (*t*-like)/123.9 (CH-12), four oxygen-bearing methine protons at *δ*_H_ 3.30 (dd, *J* = 11.4, 3.8)/90.3 (CH-3), 4.74 (br s)/67.4 (CH-16), 6.40 (d, *J* = 9.9)/81.0 (CH-21), and 4.48/71.0 (C-22), and one primary alcoholic function at *δ*_H_/*δ*_C_ 4.26/66.2 (CH_2_-28). The assignments of their position were determined by HMBC cross-peaks at *δ*_H_/*δ*_C_ 1.19 (H_3_-23)/90.3 (C-3), 1.12 (H_3_-29)/81.0 (C-21), and 4.48 (H-22)/66.2 (C-28) (Figure 2) and by COSY cross-peaks at *δ*_H_/*δ*_H_ 6.40 (H-21)/4.48 (H-22) and 1.71 (H-15)/4.74 (H-16). The configurations of C-3, C-16, C-21, and C-22 were characterized through analysis of the ROESY spectrum; ROESY cross-peaks at *δ*_H_/*δ*_H_ 1.19 (H_3_-23 α-equatorial)/3.30 (H-3 α-axial), 0.98 (H_3_-26 β-axial)/4.74 (H-16 β-equatorial), 1.12 (H_3_-29 α-equatorial)/6.40 (H-21 α-axial), and 1.33 (H_3_-30 β-axial)/4.48 (H-22 β-axial) validated the 3β-OH, 16α-OH, 21β-OH, and 22α-OH configurations (Figure 3). On the basis of all these conclusions, the aglycone of **1** was identified as (3β,16α,21β,22α)-3,16,21,22,28-pentol-olean-12-ene, named barringtogenol C, in full agreement with literature data [5].

The deshielded chemical shift of CH-21 at *δ*_C_/*δ*_H_ 81.0/6.40 ppm and CH_2_-28 at *δ*_C_/*δ*_H_ 66.2/4.26 ppm suggested an acylation at these positions. This was confirmed by the HMBC cross-peaks at *δ*_H_/*δ*_C_ 6.40 (H-21)/168.8 (Ang-1) and *δ*_H_/*δ*_C_ 4.26 (H_2_-28)/171.0 (Ac-1). At the C-21 position, the substituent was composed of two vinylic methyl groups at 2.01 (s) and 2.07 (d, *J* = 6.7 Hz) ppm, which correlated in the HMBC spectrum with one ethylenic quaternary carbon at 129.0 and an ethylenic methine carbon at 136.2 ppm. These data revealed an angeloyl group acylating the C-21 position (Table 1) [5]. The NMR signals for the acylating group at C-28 were in accordance with an acetyl function. Thus, the structure of the acylated aglycone was elucidated as 21-*O*-angeloyl-28-*O*-acetylbarringtogenol C.

The presence of three sugar moieties in **1** was evidenced by the ^1^H-NMR spectrum which displayed signals of three anomeric protons at *δ*_H_ 4.85 (d, *J* = 7.3 Hz), 5.38 (d, *J* = 7.6 Hz), and 5.65 (d, *J* = 7.0 Hz), giving correlations in the HSQC spectrum with three anomeric carbons at *δ*_C_ 104.6, 104.2, and 103.0, respectively (Table 2). Complete assignments of each sugar were achieved by extensive 1D- and 2D-NMR analyses, allowing the identification of one GlcA, one Glc, and one Ara unit. In the HMBC spectrum, correlations at *δ*_H_/*δ*_C_ 4.85 (GlcA-1)/90.3 (C-3), 5.65 (Glc-1)/77.9 (GlcA-2), and 5.38 (Ara-1)/84.8 (GlcA-3) revealed the structure of the sequence linked at the C-3 position as 3-*O*-Glc-(1→2)-[Ara-(1→3)]-GlcA. This was confirmed by the ROESY cross-peaks at δ_H_/δ_H_ 4.85 (GlcA-1)/3.30 (H-3), 5.65 (Glc-1)/4.52 (GlcA-2), and 5.38 (Ara-1)/4.40 (t, *J* = 8.8 Hz, GlcA-3). The structure of compound **1** was, thus, established as 3-*O*-β-d-glucopyranosyl-(1→2)-[α-l-arabinopyranosyl-(1→3)]-β-d-glucuronopyranosyl-21-*O*-angeloyl-28-*O*-acetylbarringtogenol C.

The HR-ESI-MS of compound **2** was the same as compound **1**, with a molecular formula of C_54_H_84_O_22_. Extensive 2D-NMR analysis (Table 1) showed that **2** and **1** possess the same acylated aglycone and differ only by the osidic part. In the oligosaccharidic chain, the Glc unit in **1** is replaced by a Gal unit in **2**. The HMBC correlation at *δ*_H_/*δ*_C_ 5.49 (d, *J* = 7.6 Hz, Gal-1)/78.8 (GlcA-2) and the ROESY correlation at *δ*_H_/*δ*_H_ 5.49 (Gal-1)/4.48 (GlcA-2) confirmed the 3-*O*-β-d-galactopyranosyl-(1→2)-[α-l-arabinopyranosyl-(1→3)]-β-d-glucuronopyranosyl sequence. Thus, the structure of **2** was elucidated as 3-*O*-β-d-galactopyranosyl-(1→2)-[α-l-arabinopyranosyl-(1→3)]-β-d-glucuronopyranosyl-21-*O*-angeloyl-28-*O*-acetylbarringtogenol C. Natural similar compounds of **1**, **2** with acylated 3-*O*-glucuronopyranosylbarringtogenol C derivatives have already been isolated from a “variegatum” cultivar of *P*. *tenuifolium* [5].

**Figure 4:**
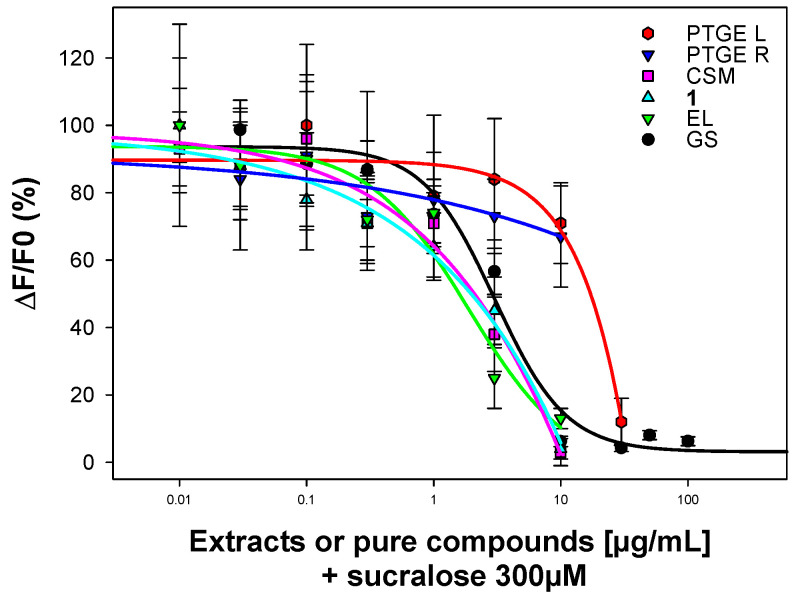
Sucralose responses of hTAS1R2/hTAS1R3 treated by PTGE L, PTGE R, CSM, **1**, EL, and GS.

Some saponins are known for their sweet taste such as glycyrrhizin from licorice, as well as for their sweet inhibitor activity such as gymnemic acids (GS), which correspond to a saponin mixture from *Gymnema sylvestre* (Apocynaceae) [11,12]. The sweet taste is mediated by the TAS1R2/TAS1R3 receptor found in the oral cavity and in various extraoral tissues such as the pancreas, brain, and bones [13]. The aglycone of GS named gymnemagenin is a polyhydroxylated oleanane-type derivative (3β,16β,21β,22α)3,16,21,22,23,28-hexol-olean-12-ene. This aglycone differs from barringtogenol C (3β,16α,21β,22α)-3,16,21,22,28-pentol-olean-12-ene by only one hydroxylation at the C-23 position. Moreover, GS structures possess acylation at the 21 and 22 positions, in addition to a 3-*O*-heterosidic linkage with a glucuronopyranosyl moiety. The interaction between this glucuronopyranosyl residue and the transmembrane domain of hTAS1R3 has already been described [14]. According to these similarities between the isolated compounds from *Pittosporum tenuifolium* “Green Elf” and GS, an aqueous ethanolic extract of the leaves (PTGE L) and roots (PTGE R), a crude saponin mixture (CSM), the pure compounds **1**, and Eryngioside L (EL) were tested as TAS1R2/TAS1R3 inhibitors. Human embryonic kidney HEK293T-Gα16gust44 cells were transiently transfected with two plasmids coding for hTAS1R2 and hTAS1R3 subunits, respectively. Then, the cellular responses of cells to sucralose were measured by calcium mobilization assay after application of increasing concentrations of GS or plant extracts. Firstly, PTGE L, PTGE R, CSM, **1**, and EL were evaluated for stimulation of the sweet taste receptor, with sucralose as a positive control (EC_50_ = 52 ± 7 μM) (Supplementary Figure S11). None of them showed activation of the TAS1R2/TAS1R3. Then, the same compounds and GS (isolated from a commercial product; see Section 3) were tested for inhibition of the sucralose response (Figure 4). At higher concentrations, between 3 and 10 μg/mL, a decrease in sucralose response was observed, but this may be linked to a toxic effect. Actually, this toxicity could be observed for the control cells. On the contrary, GS showed an inhibitory effect at IC_50_ = 2.97 ± 0.64 μg/mL (Supplementary Figure S12), according to results previously published [15]. From a structure/activity relationship point of view, the lack of toxic effects of GS compared with the toxicity of molecules isolated from *Pittosporum tenuifolium* “Green Elf” could be related to the presence of a secondary alcoholic function at the C-23 position of gymnemagenin. This conclusion needs to be proven by further tests with saponins possessing gymnemagenin-type aglycones.

## 3. Materials and Methods

### 3.1. General Experimental Procedures

Optical rotation values were recorded on an AA-10R automatic polarimeter (Optical Activity LTD, Ramsey, Cambridgeshire, PE26 1NF, UK). The 1D and 2D spectra (^1^H- and ^13^C-NMR, ^1^H–^1^H COSY, TOCSY, ROESY, HSQC, and HMBC) were performed using an NMR Inova 600 MHz spectrometer (Agilent Technologies) equipped with 3 mm triple resonance inverse and 3 mm dual broadband probeheads. Spectra were recorded in pyridine-*d*_5_. Solvent signals were used as internal standard (pyridine-*d*_5_: *δ*_H_ = 7.21, *δ*_C_ = 123.5 ppm), and all spectra were recorded at T = 35 °C. Pulse sequences were taken from the Varian pulse sequence library (gCOSY; gHSQCAD and gHMBCAD with adiabatic pulses CRISIS-HSQC and CRISIS-HMBC). TOCSY spectra were acquired using DIPSI spin-lock and a 150 ms mixing time. Mixing time in ROESY experiments was 300 ms. Coupling constants (*J*) were measured in Hz. HR-ESI-MS and ESI-MS were carried out on a Bruker micrOTOF II mass spectrometer. For the extractions, an ultrasound water bath apparatus (S15 Elmasonic 35W) was used. The compound isolation was carried out using vacuum liquid chromatography (VLC) on silica gel 60 (Sigma-Aldrich, St. Louis, MO, USA, 63–200 μm) and column chromatography (CC) on Sephadex LH-20 (550 mm × 20 mm, GE Healthcare Bio-Sciences AB). Medium-pressure liquid chromatography (MPLC) was performed on a silica gel 60 (15–40 μm, Merck, Rahway, NJ, USA) and a reversed-phase RP-18 silica gel (75–200 μm, Silicycle), with a Gilson M 305 pump (25 SC head pump, M 805 manometric module), a Büchi glass column (460 mm × 25 mm and 460 mm × 15 mm), and a Büchi precolumn (110 mm × 15 mm). HPLC was performed on a 1260 Agilent instrument, equipped with a degasser, a quaternary pump, an autosampler, and a UV detector at 210 nm. Analytical separations were carried out on an RP-18 column (250 mm × 4.6 mm id, 5 μm; Phenomenex LUNA) at room temperature and protected by a guard column. Solvent A was 0.01% (*v*/*v*) aqueous trifluoroacetic acid, and solvent B was acetonitrile, 1 mL/min, with detection at 210 nm, going from 30% to 80% B in 30 min. Semi-preparative (1/2 prep) separations were carried out on a RP-18 column (250 mm × 10 mm id, 5 μm; Phenomenex LUNA) at room temperature and protected by a guard column. Solvent A was 0.01% (*v*/*v*) aqueous trifluoroacetic acid, and solvent B was acetonitrile, going from 40% to 90% B in 30 min, at 5 mL/min. The injection volume was 200 μL. Thin-layer chromatography (TLC, Silicycle) and high-performance thin-layer chromatography (HPTLC, Merck) were carried out on precoated silica gel plates 60F_254_, with solvent system CHCl_3_/MeOH/H_2_O (70:30:5). The spray reagent for saponins was vanillin (1% vanillin (Sigma-Aldrich) in EtOH/H_2_SO_4_, 50:1).

### 3.2. Plant Material

The cultivar “Green Elf” of *Pittosporum tenuifolium* Banks & Sol. ex Gaertn. (Pittosporaceae) was purchased in 2017 from Jardiland^®^ (Chenôve, France). A voucher specimen (N° 2016/05/05) was deposited in the herbarium of the Laboratory of Pharmacognosy, Université de Bourgogne Franche-Comté, Dijon, France.

### 3.3. Extraction and Isolation

Dried powdered leaves of *P. tenuifolium* “Green Elf” (29 g) were submitted to ultrasonic-assisted extraction at room temperature, five times, 30 min each, with a mixture of EtOH–H_2_O (75:35, 5 × 225 mL). After evaporation of the solvent under vacuum, 7.9 g of extract was obtained. An aliquot of 3 g was fractionated by vacuum liquid chromatography (VLC) on silica gel (CHCl_3_/MeOH/H_2_O 80:20:2; 70:30:5; 60:32:7; 0:100:0), yielding three fractions F1 to F3. F2 (1 g) was submitted to column chromatography (Sephadex LH-20) yielding two fractions, F2.1 and F2.2. Fraction F2.1 (285 mg) was then separated by successive MPLC on silica gel (CHCl_3_/MeOH/H_2_O 70:30:5 and 60:32:7, 2.5 mL/min) and on reverse-phase silica gel RP-18 (MeOH/H_2_O 40:60 for 15 min, 45:55 for 15 min, and 50:50 for 60 min), yielding the final fractions. Each final fraction was analyzed by analytical HPLC. The chromatograms allowed to determine three main peaks at *tr*_1_ 16.0 min, *tr*_2_ 16.7 min, and *tr*_3_ 16.8 min. A final purification by semi-preparative HPLC resulted in the isolation of **1** (5.2 mg, *tr*_3_), **2** (3.7 mg, *tr*_2_), and the known Eryngioside L (5.3 mg, *tr*_1_).

Dried powdered roots of *P. tenuifolium* “Green Elf” (227 g) were submitted to an ultrasonic-assisted extraction, three times, 20 min each, in 1 L of EtOH/H_2_O 75:35 as solvent. After evaporation of the solvent under vacuum, 14 g of extract was obtained and then fractionated according to the same protocol used for the leaves, yielding the known Sandrosaponin X (5.0 mg).

3-*O*-β-d-Glucopyranosyl-(1→2)-[α-l-arabinopyranosyl-(1→3)]-β-d-glucuronopyranosyl-21-*O*-angeloyl-28-*O*-acetylbarringtogenol C (**1**). White amorphous powder. [α]^25^_D_ = −9.5 (*c* 0.12, MeOH). For ^1^H-NMR (pyridine-*d*_5_, 600 MHz) and ^13^C-NMR (pyridine-*d*_5_, 150 MHz) data, see Table 1 and Table 2. HR-ESI-MS (positive-ion mode) *m*/*z*: 1107.5359 [M + Na]^+^ (calculated for C_54_H_84_NaO_22_, 1107.5352).

3-*O*-β-d-galactopyranosyl-(1→2)-[α-l-arabinopyranosyl-(1→3)]-β-d-glucuronopyranosyl-21-*O*-angeloyl-28-*O*-acetylbarringtogenol C (**2**). White amorphous powder. [α]^25^_D_ = −13.7 (*c* 0.15, MeOH). For ^1^H-NMR (pyridine-*d*_5_, 600 MHz) and ^13^C-NMR (pyridine-*d*_5_, 150 MHz) data, see Table 1 and Table 2. HR-ESI-MS (positive-ion mode) *m*/*z*: 1107.5359 [M + Na]^+^ (calculated for C_54_H_84_NaO_22_, 1107.5352).

### 3.4. Acid Hydrolysis and Absolute Configuration Determination

An aliquot (180 mg) of a rich saponin fraction was hydrolyzed with 2 N aqueous CF_3_COOH (25 mL) for 3 h at 95 °C. After extraction with CH_2_Cl_2_ (3 × 15 mL), the aqueous layer was evaporated to dryness with H_2_O until neutral to give the sugar residue (64 mg). Glucuronic acid, glucose, galactose, and arabinose were identified by comparison with authentic samples by TLC using CH_3_COOEt/CH_3_COOH/CH_3_OH/H_2_O (65:25:15:15). After purification of these sugars by prep-TLC in the same solvent, the optical rotation of each purified sugar was measured as follows: d-galactose, *R*_f_ = 0.47, [α]^25^_D_ + 130 (*c* 0.2, H_2_O), l-arabinose, *R*_f_ = 0.54, [α]^25^_D_ + 170 (*c* 0.2, H_2_O), d-glucuronic acid, *R*_f_ = 0.24, [α]^25^_D_ + 15 (*c* 0.2, H_2_O), and d-glucose, *R*_f_ = 0.50, [α]^25^_D_ + 110 (*c* 0.2, H_2_O).

### 3.5. Bioactivity Assay

For functional experiments on the sweet taste receptor, HEK293T cells stably expressing the chimeric G-protein subunit Gα16gust44 were seeded into 96-well plates as previously described [16]. Then, 24 h after seeding, cells were transiently transfected with hTAS1R2 and hTAS1R3 cDNAs, cloned into pcDNA6 and pcDNA4 vectors (Life Technologies, Carlsbad, CA, USA), respectively, with plasmid pCMV-GCaMP5G (Addgene) used as a genetically encoded calcium indicator. HEK293T-Gα16gust44 cells transfected with an empty vector served as a negative control. Then, after 24 h, cells were washed two times with C1 solution (130 mM NaCl, 5 mM KCl, 10 mM Hepes, 2 mM CaCl_2_, 5 mM sodium pyruvate, pH 7.4). Test substances were initially solubilized in DMSO, followed by in C1 solution for further dilution, before being subjected to 10 min stimulation on cells. After washing with C1 buffer, cells were stimulated by automatic injection of 300 µM sucralose, and changes in intracellular calcium levels were measured at 510 nm in a Fluorometric Imaging Plate Reader (FLIPR, Molecular Devices) after excitation at 488 nm. For data analysis of dose–response curves, signals of wells receiving the same treatment were averaged, the fluorescent signal of mock cells was subtracted from receptor-transfected cells, and the net signal was normalized to background (ΔF/F_0_, F_0_ fluorescence light before stimulus application). For calculation of the half-maximal effective concentration EC_50_ and half-maximal inhibitory concentration IC_50_ values, ΔF/F_0_ was plotted against concentration of the test substance using a four-parameter logistic equation [f(x) = min + (max − min)/(1 + (*x*/EC_50_)^nH^)] with curves fitting of Sigma Plot software.

A total of 15.9 g of tablets of “SMART *Gymnena sylvestre* standardised extract 75% gymnemic acids” were dissolved in EtOH/H_2_O, stirred for 5h, and then submitted to ultrasound-assisted extraction at room temperature (24 KHz, 200 W, 30 min). After filtration and evaporation of the solvent, the crude extract (9 g) was submitted to vacuum liquid chromatography (VLC) over silica gel (CHCl_3_/MeOH/H_2_O 60:32:7), yielding five fractions Fr1 to Fr5. Fr3 (1.4 g), rich in saponin, was submitted to flash chromatography over silica gel (CHCl_3_/MeOH/H_2_O 80:20:2, 70:30:5), yielding three fractions Fr3.1 to Fr3.3. Fr3.2 (880 mg) was fractionated by VLC on RP-18 (MeOH/H_2_O 0:100, 100:0), yielding a fraction rich in gymnemic acids (GS).

## 4. Conclusions

Four oleanane-type glycosides were isolated from a horticultural cultivar “Green Elf” of the endemic *Pittosporum tenuifolium* (Pittosporaceae) from New Zealand, with two previously undescribed: 3-*O*-β-d-glucopyranosyl-(1→2)-[α-l-arabinopyranosyl-(1→3)]-β-d-glucuronopyranosyl-21-*O*-angeloyl-28-*O*-acetylbarringtogenol C and 3-*O*-β-d-galactopyranosyl-(1→2)-[α-l-arabinopyranosyl-(1→3)]-β-d-glucuronopyranosyl-21-*O*-angeloyl-28-*O*-acetylbarringtogenol C. The inhibitory activities on the sweet taste TAS1R2/TAS1R3 receptor of an aqueous ethanolic extract of the leaves and roots, a crude saponin mixture, the pure compound **1**, and Eryngioside L were evaluated. None of them showed clear activity, but exhibited real toxicity. The lack of toxic effects of gymnemic acids, compared with the toxicity of the molecules isolated from *Pittosporum tenuifolium* “Green Elf”, could be related to the presence of a secondary alcoholic function at the C-23 position of gymnemagenin.

## Figures and Tables

**Table 1:** ^1^H (600 MHz) and ^13^C (150 MHz) NMR data of the aglycone moieties of compounds **1** and **2** in pyridine-*d*_5_ (*δ* in ppm, *J* in parentheses in Hz) ^a^.

Position	1		2	
	*δ*_C_	*δ*_H_	*δ*_C_	*δ*_H_
1	38.6	0.84 1.38	38.6	0.85 1.38
2	26.1	1.94 m 2.33 m	26.1	1.92 2.34 m
3	90.3	3.30 dd (11.4, 3.8)	89.9	3.28
4	39.4	-	39.4	-
5	55.5	0.74 d (11.4)	55.6	0.74 br d (11.4)
6	18.2	1.30 1.49 m	18.2	1.30 1.48 m
7	32.8	1.30 1.56 m	32.9	1.30 1.56 m
8	39.7	-	39.7	-
9	46.7	1.68 m	46.7	1.68
10	36.5	-	36.5	-
11	23.6	1.86 1.86	23.6	1.84 1.84
12	123.9	5.50 *t*-like	124.0	5.49
13	142.2	-	142.4	-
14	41.5	-	41.5	-
15	34.1	1.71 m 1.90	34.2	1.71 1.90
16	67.4	4.74 br s	67.4	4.74 br s
17	47.0	-	47.0	-
18	40.3	2.85 dd (13.2, 3.1)	40.3	2.86 br d (13.7)
19	46.8	1.44 m 3.08 t (11.7)	46.8	1.45 m 3.09 t (11.6)
20	35.8	-	35.8	-
21	81.0	6.40 d (9.9)	81.0	6.41 d (10.0)
22	71.0	4.48	71.0	4.48
23	27.6	1.19 s	27.7	1.27 s
24	16.4	1.09 s	16.5	1.14 s
25	15.4	0.82 s	15.4	0.81 s
26	16.8	0.98 s	16.8	0.97 s
27	27.1	1.81 s	27.1	1.81 s
28	66.2	4.26 4.26	66.2	4.26 4.26
29	29.5	1.12 s	29.5	1.11 s
30	19.9	1.33 s	19.9	1.33 s
	Ang at C-21		Ang at C-21	
1′	168.8	-	168.7	-
2′	129.0	-	129.1	-
3′	136.2	5.98 q (7.3)	136.1	5.97 q (6.8)
4′	15.7	2.07 d (6.7)	15.7	2.06 d (6.8)
5′	20.8	2.01 s	20.8	2.01 s
	Ac at C-28		Ac at C-28	
1″	171.0	-	170.9	-
2″	20.5	2.06 s	20.5	2.04 s

^a^ Overlapped signals are reported without designated multiplicity. *δ* in ppm; *J* in parentheses in Hz.

**Table 2:** ^1^H (600 MHz) and ^13^C (150 MHz) NMR data of the sugar moieties of compounds **1** and **2** in pyridine-*d*_5_ (*δ* in ppm, *J* in parentheses in Hz) ^a^.

Position	1		2	
	*δ*_C_	*δ*_H_	*δ*_C_	*δ*_H_
	3-*O*-GlcA		3-*O*-GlcA	
1	104.6	4.85 d (7.3)	104.7	4.85 d (7.6)
2	77.9	4.52	78.8	4.48
3	84.8	4.40 t (8.8)	84.9	4.36
4	71.9	4.24	71.3	4.22
5	76.9	4.33 m	76.5	4.32
6	Nd		Nd	
	Glc		Gal	
1	103.0	5.65 d (7.0)	103.8	5.49 d (7.6)
2	75.8	4.05 t (8.4)	73.2	4.46
3	77.9	4.27	74.7	4.13
4	72.0	3.99 dd (9.3, 8.7)	69.6	4.49
5	78.2	3.92 m	76.5	3.92 m
6	62.9	4.23 4.58 d (10.8)	61.7	4.36 4.43
	Ara		Ara	
1	104.2	5.38 d (7.6)	104.2	5.33 d (7.6)
2	72.4	4.47	72.4	4.47
3	74.0	4.16 dd (9.3, 2.6)	74.1	4.13
4	69.1	4.26	69.2	4.26
5	66.9	3.78 d (12.0) 4.36	67.0	3.74 d (12.3) 4.38

^a^ Overlapped signals are reported without designated multiplicity. Nd: not determined.

## Supplementary Materials

The following are available online: Figure S1. HSQC spectrum of compound **1**; Figure S2. HMBC spectra of compound **1**; Figure S3. COSY spectrum of compound **1**; Figure S4. TOCSY spectrum of compound **1**; Figure S5. ROESY spectrum of compound **1**; Figure S6. HSQC spectrum of compound **2**; Figure S7. HMBC spectra of compound **2**; Figure S8. COSY spectrum of compound **2**; Figure S9. TOCSY spectrum of compound **2**; Figure S10. ROESY spectrum of compound **2**; Figure S11. Activation of hTAS1R2-hTAS1R3 by sucralose; Figure S12. Inhibitory effect of GS on the response of sucralose by hTAS1R2-hTAS1R3.

## Sample Availability

Samples of PTGE L, PTGE R, and CSM extracts are available from the authors.
