# Supplementary material for: Triterpenoid Saponins from the Cultivar “Green Elf” of Pittosporum tenuifolium
Source: Molecules. 2021 Nov 11;26(22):6805. doi: 10.3390/molecules26226805 (PMC8625740; doi:10.3390/molecules26226805)
Supplement: Supplementary file 1 [file molecules-26-06805-s001.zip › molecules-1458131-supplementary.pdf]

Supporting Information

Triterpenoid saponins from the cultivar "Green Elf" of *Pittosporum tenuifolium*

David Pertuit [1], Anne-Claire Mitaine-Offer [1,*], Tomofumi Miyamoto [2], Chiaki Tanaka [2], Christine Belloir [3], Loïc Briand [3], Marie-Aleth Lacaille-Dubois [1]

[1] PEPITE EA 4267, Laboratoire de Pharmacognosie, UFR des Sciences de Santé, Université de Bourgogne Franche-Comté, BP 87900, 21079 Dijon cedex, France

[2] Graduate school of pharmaceutical Sciences, Kyushu University, Fukuoka, Japan

[3] Centre des Sciences du Goût et de l'Alimentation, AgroSup Dijon, CNRS, INRAE, Université Bourgogne Franche-Comté, 21065 Dijon cedex, France

* Correspondence: anne-claire.offer@u-bourgogne.fr; Tel. : +33-3-80-39-34-74

CONTENTS

Figure S1. HSQC spectrum of compound **1** 3
Figure S2. HMBC spectra of compound **1** 5
Figure S3. COSY spectrum of compound **1** 6
Figure S4. TOCSY spectrum of compound **1** 7
Figure S5. ROESY spectrum of compound **1** 8
Figure S6. HSQC spectrum of compound **2** 9
Figure S7. HMBC spectra of compound **2** 11
Figure S8. COSY spectrum of compound **2** 12
Figure S9. TOCSY spectrum of compound **2** 13
Figure S10. ROESY spectrum of compound 2 14
Figure S11. Activation of hTAS1R2-hTAS1R3 by sucralose........ 15
Figure S12. Inhibitory effect of GS on the response of sucralose by hTAS1R2-hTAS1R3........16

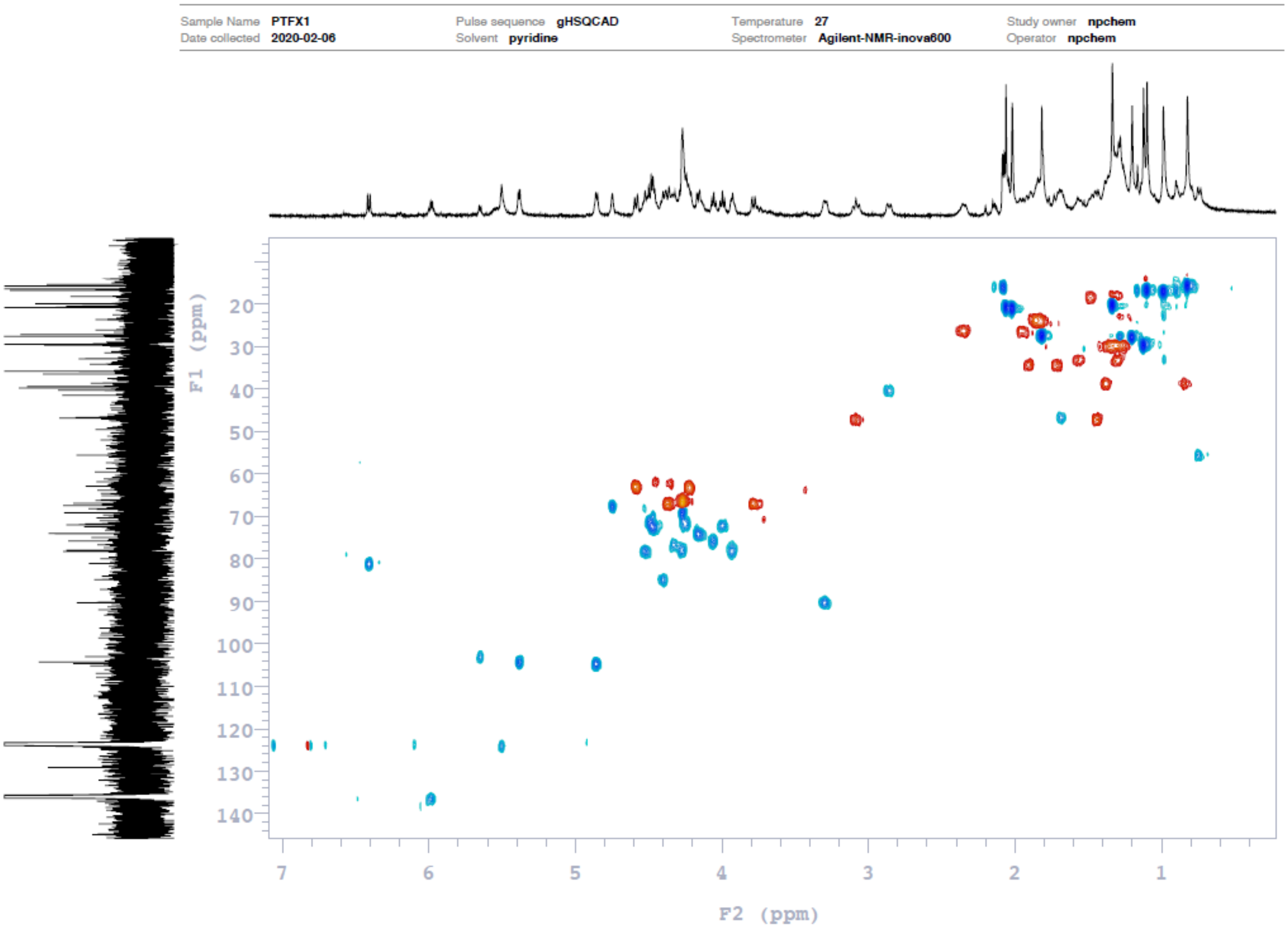

Figure S1. HSQC spectrum of compound **1**

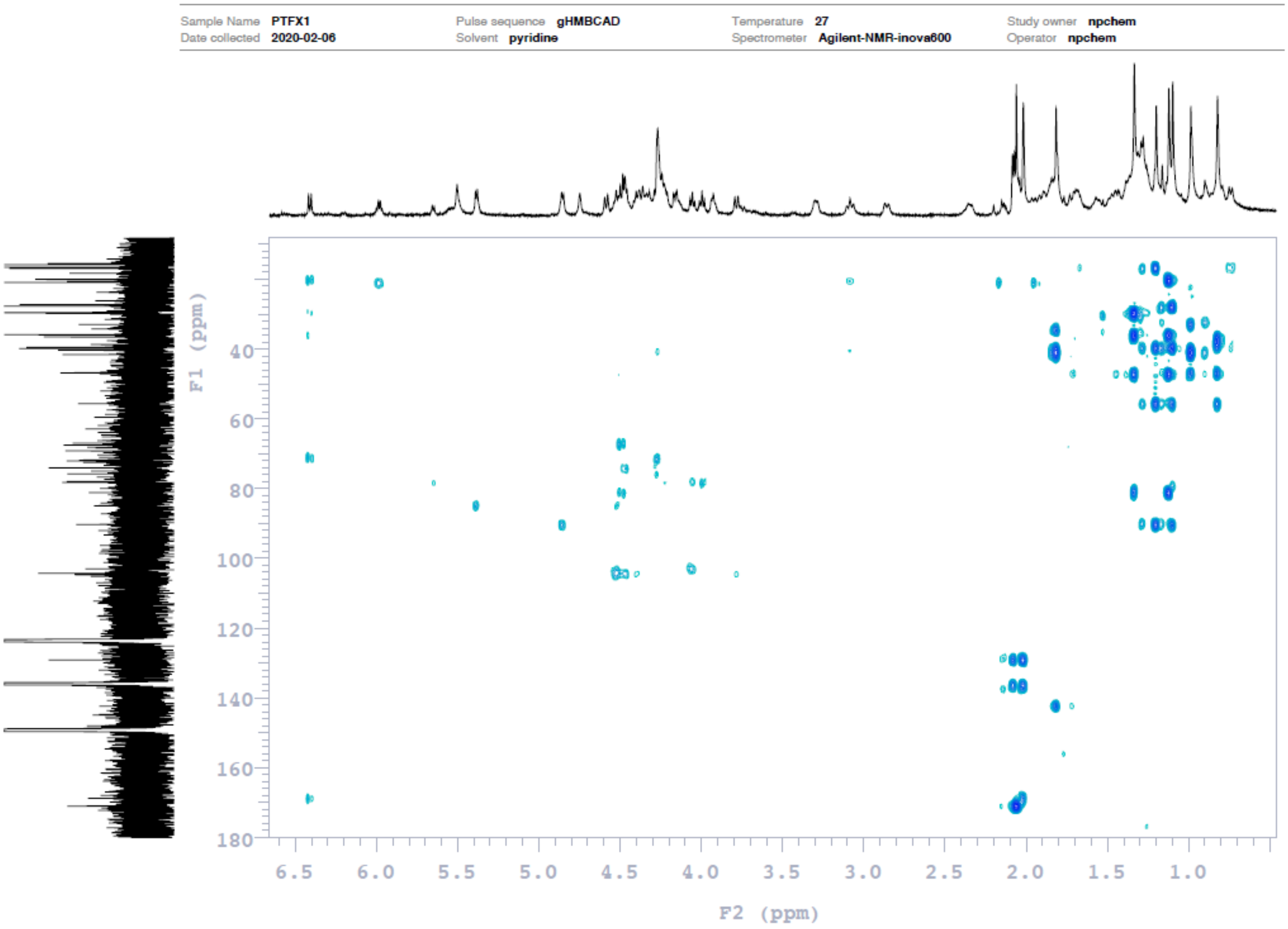

Sample Name PTFX1
Date collected 2020-02-06
Pulse sequence gHMBCAD
Solvent pyridine
Temperature 27
Spectrometer Agilent-NMR-inova600
Study owner npchem
Operator npchem
F1 (ppm)
F2 (ppm)

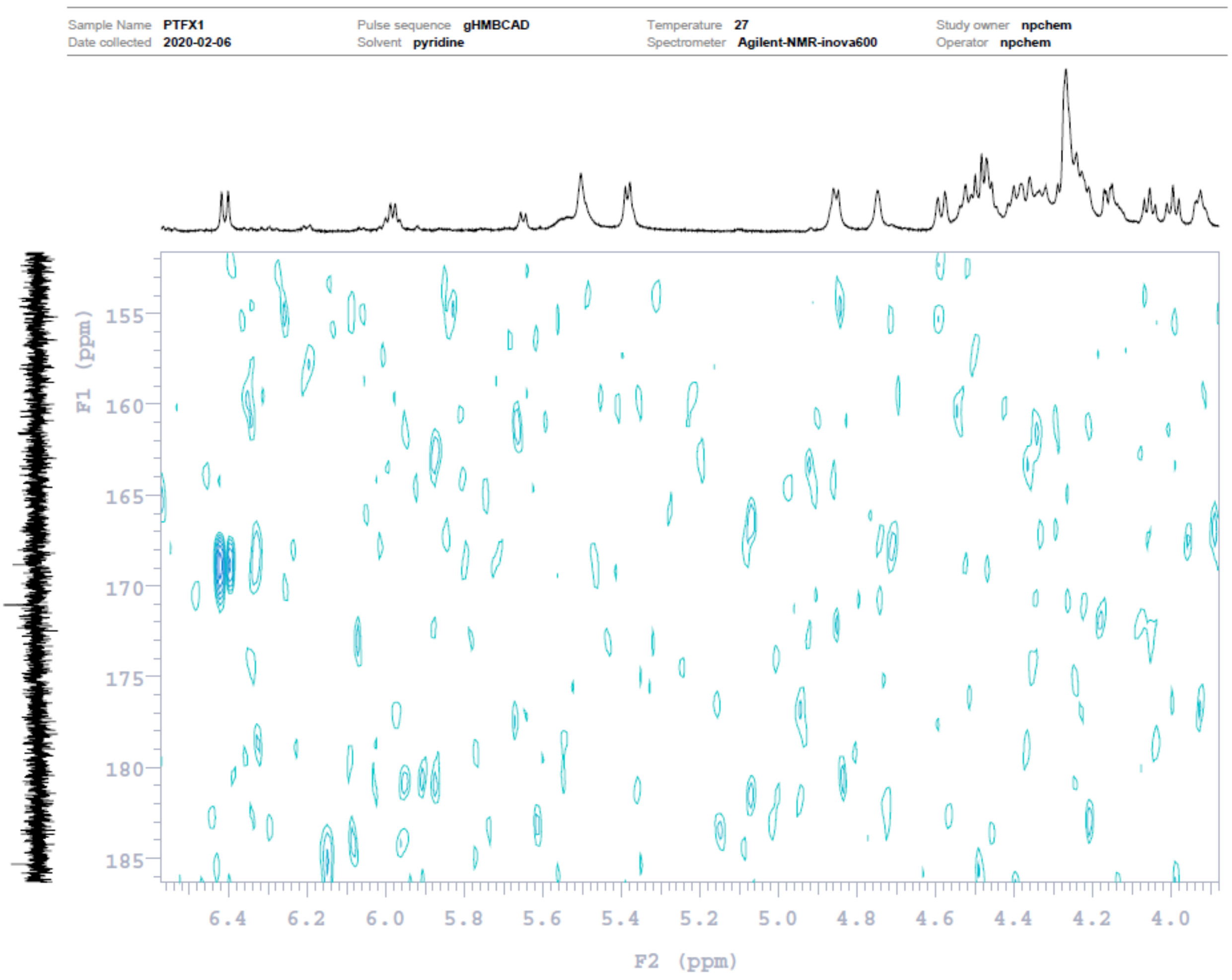

Figure S2. HMBC spectra of compound **1**

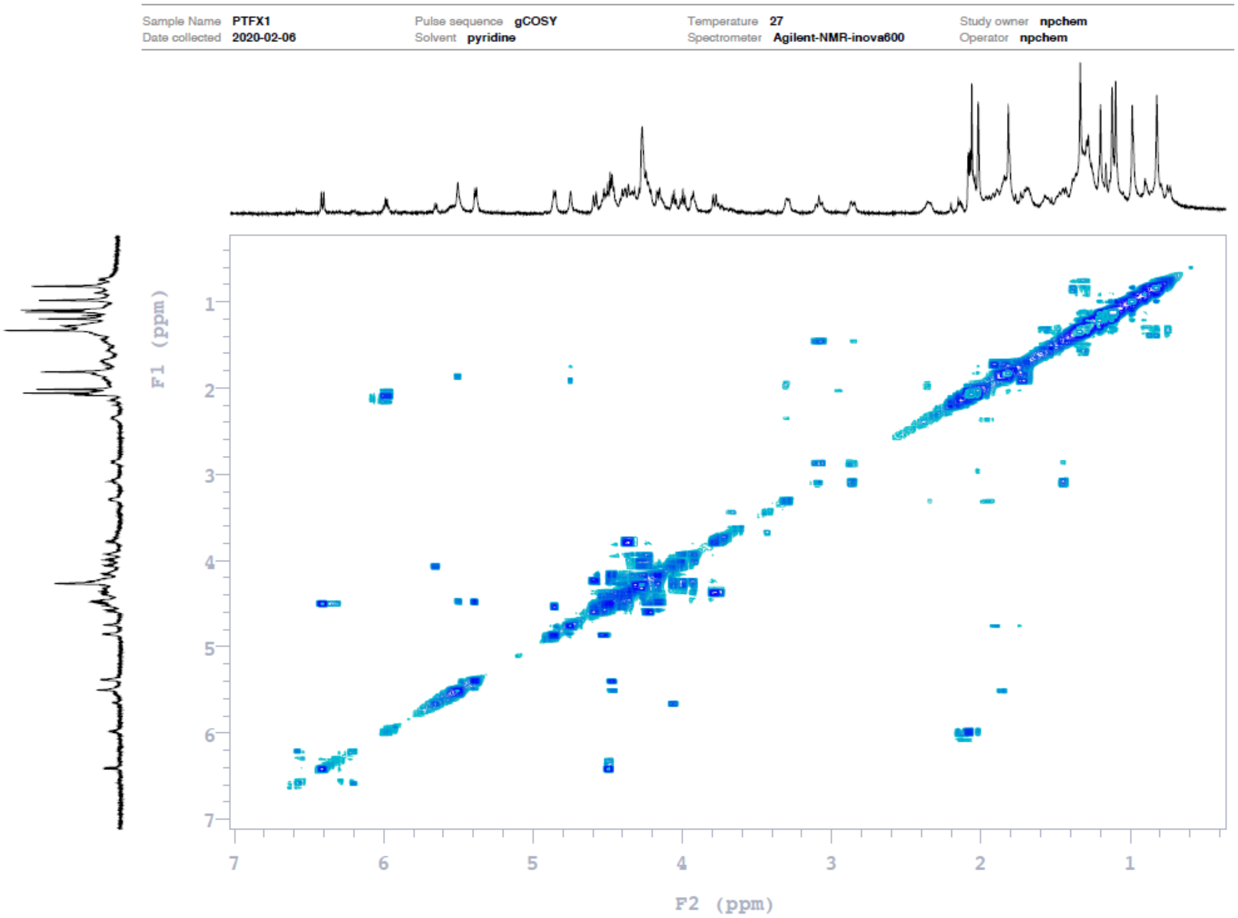

Figure S3. COSY spectrum of compound **1**

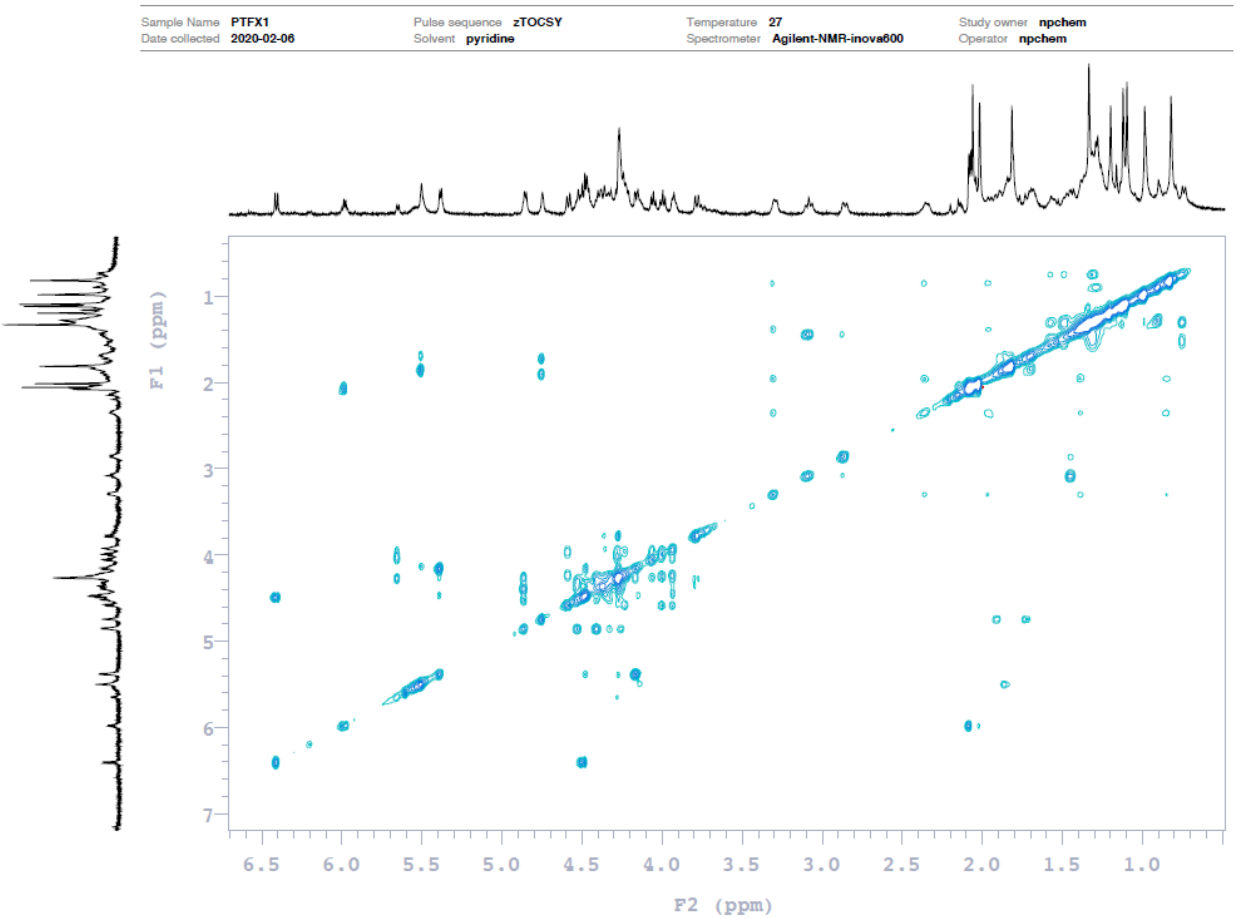

Figure S4. TOCSY spectrum of compound **1**

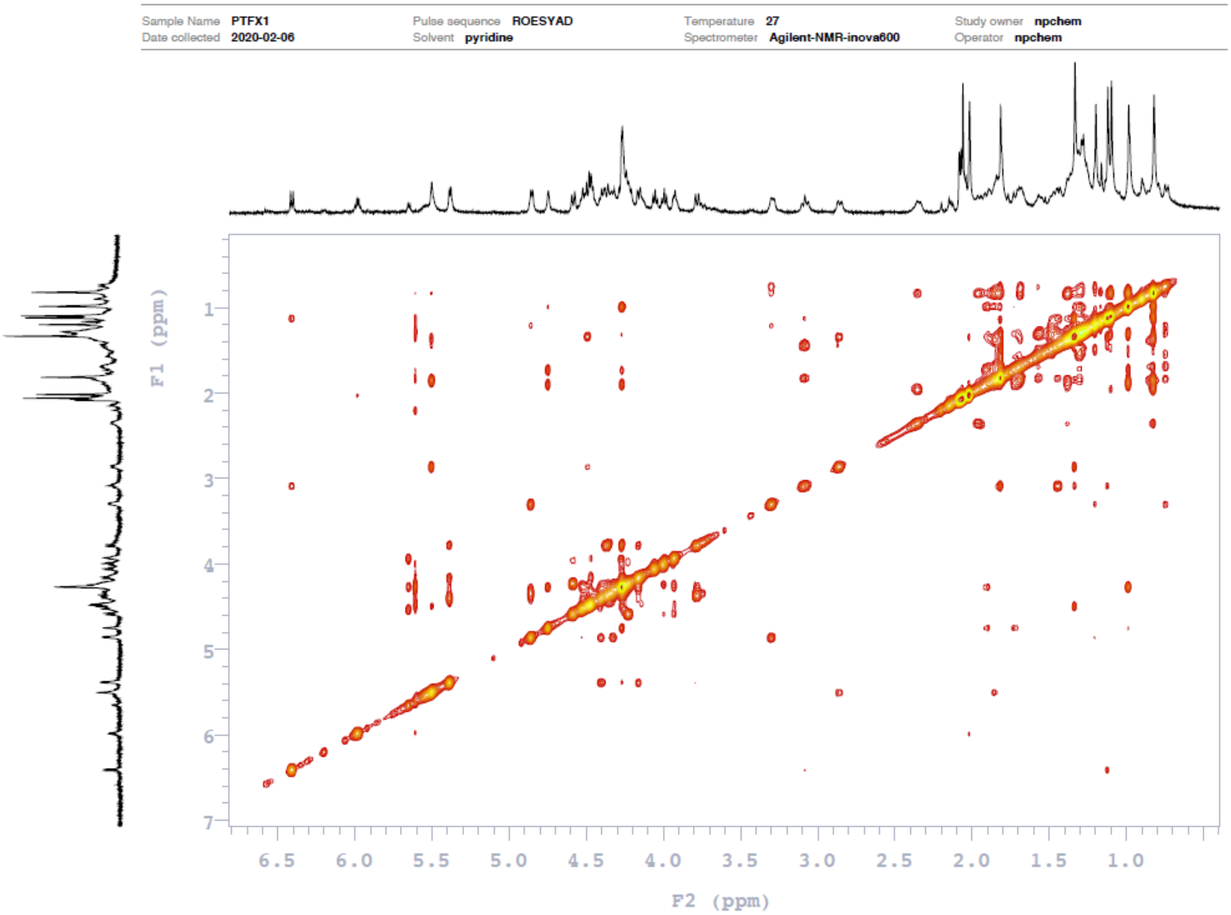

Figure S5. ROESY spectrum of compound **1**

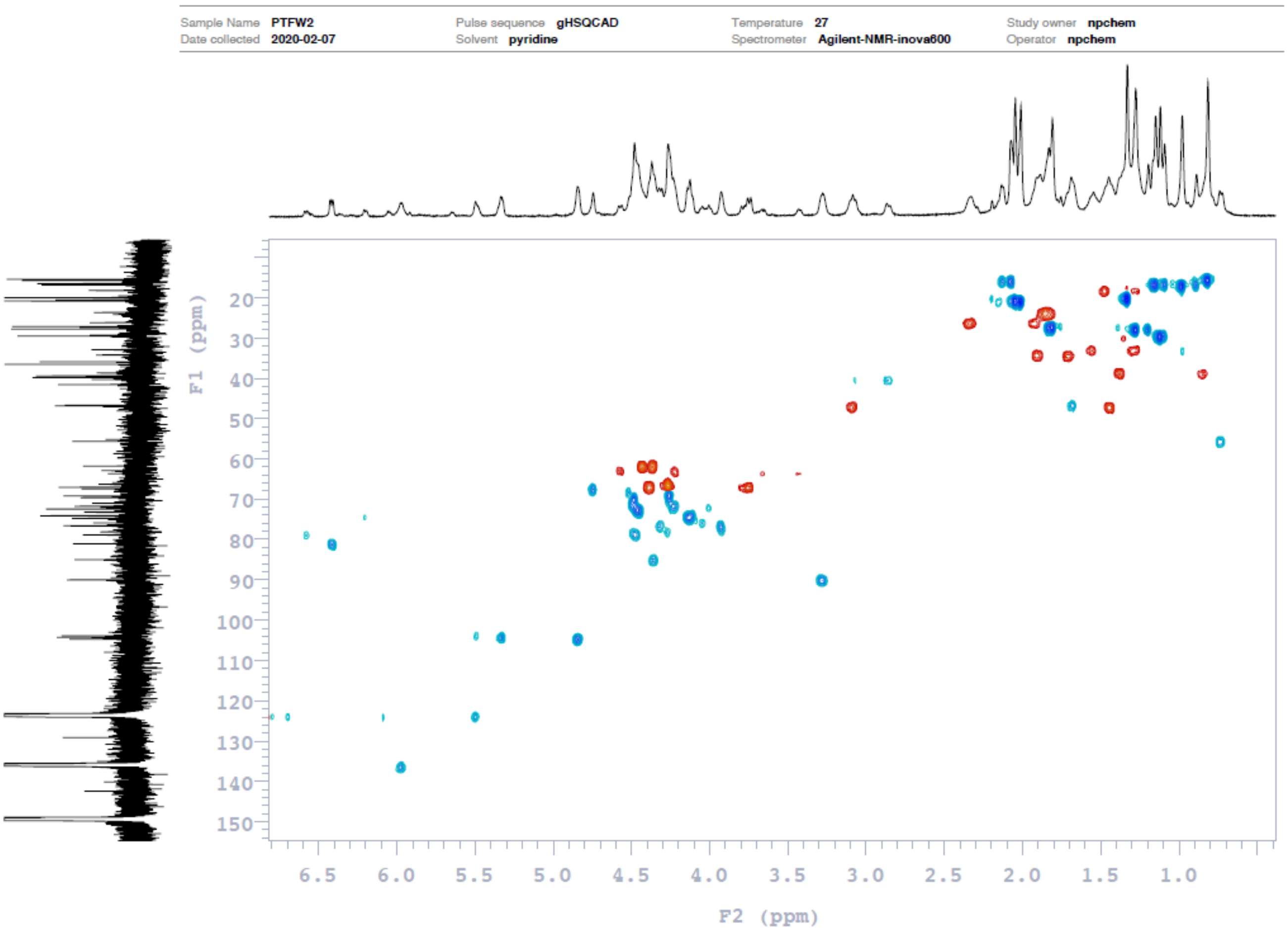

Figure S6. HSQC spectrum of compound **2**

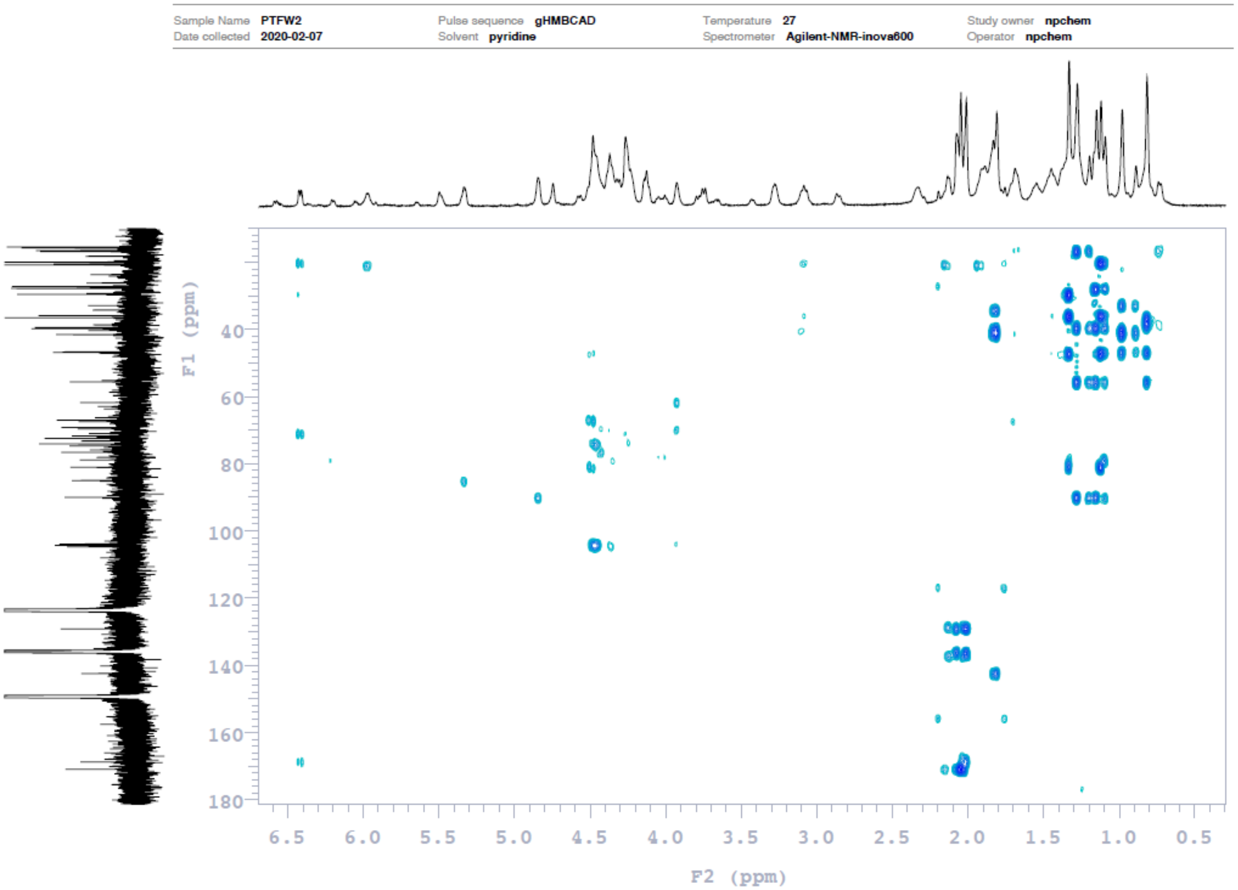
Sample Name PTFW2
Date collected 2020-02-07
Pulse sequence gHMBCAD
Solvent pyridine
Temperature 27
Spectrometer Agilent-NMR-inova600
Study owner npchem
Operator npchem
F1 (ppm)
F2 (ppm)

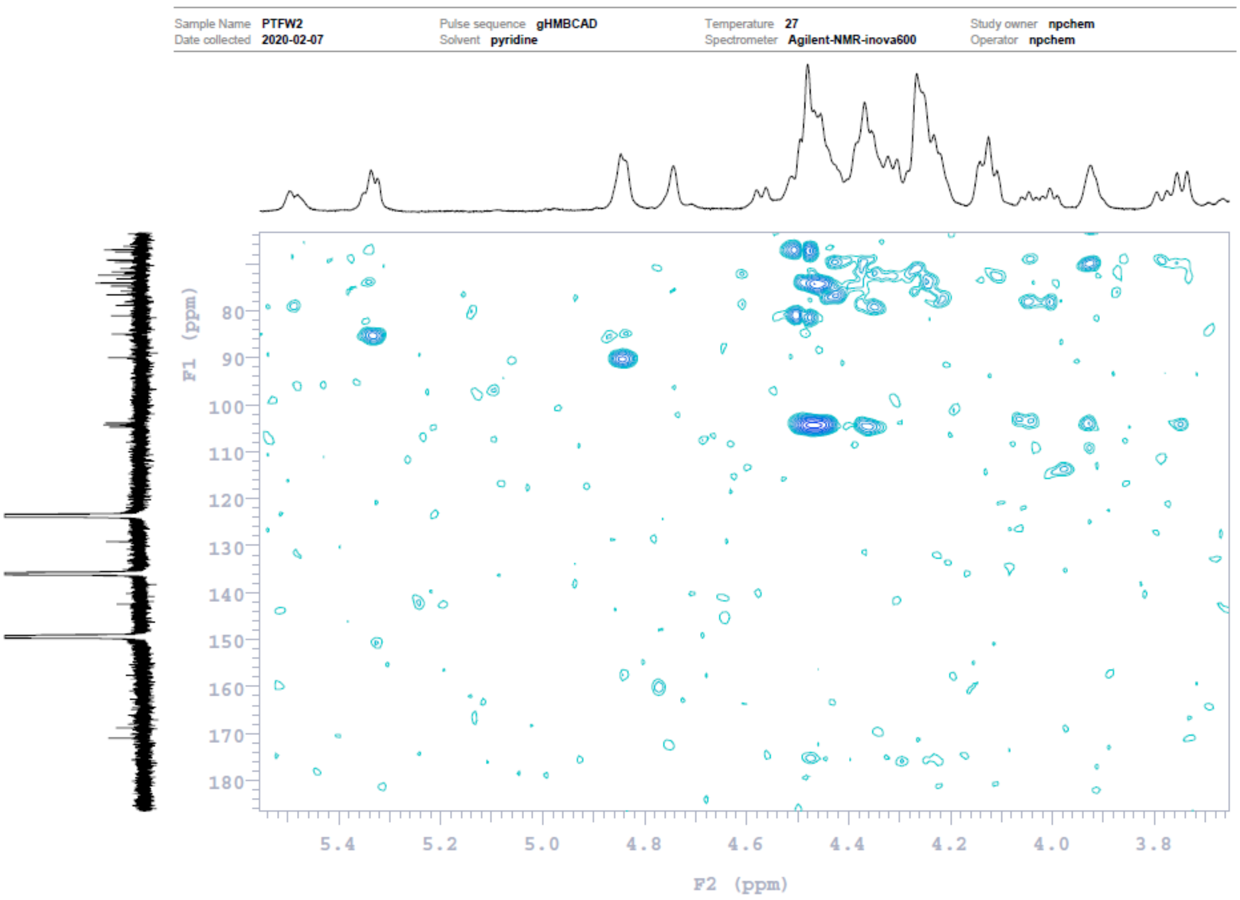

Figure S7. HMBC spectra of compound **2**

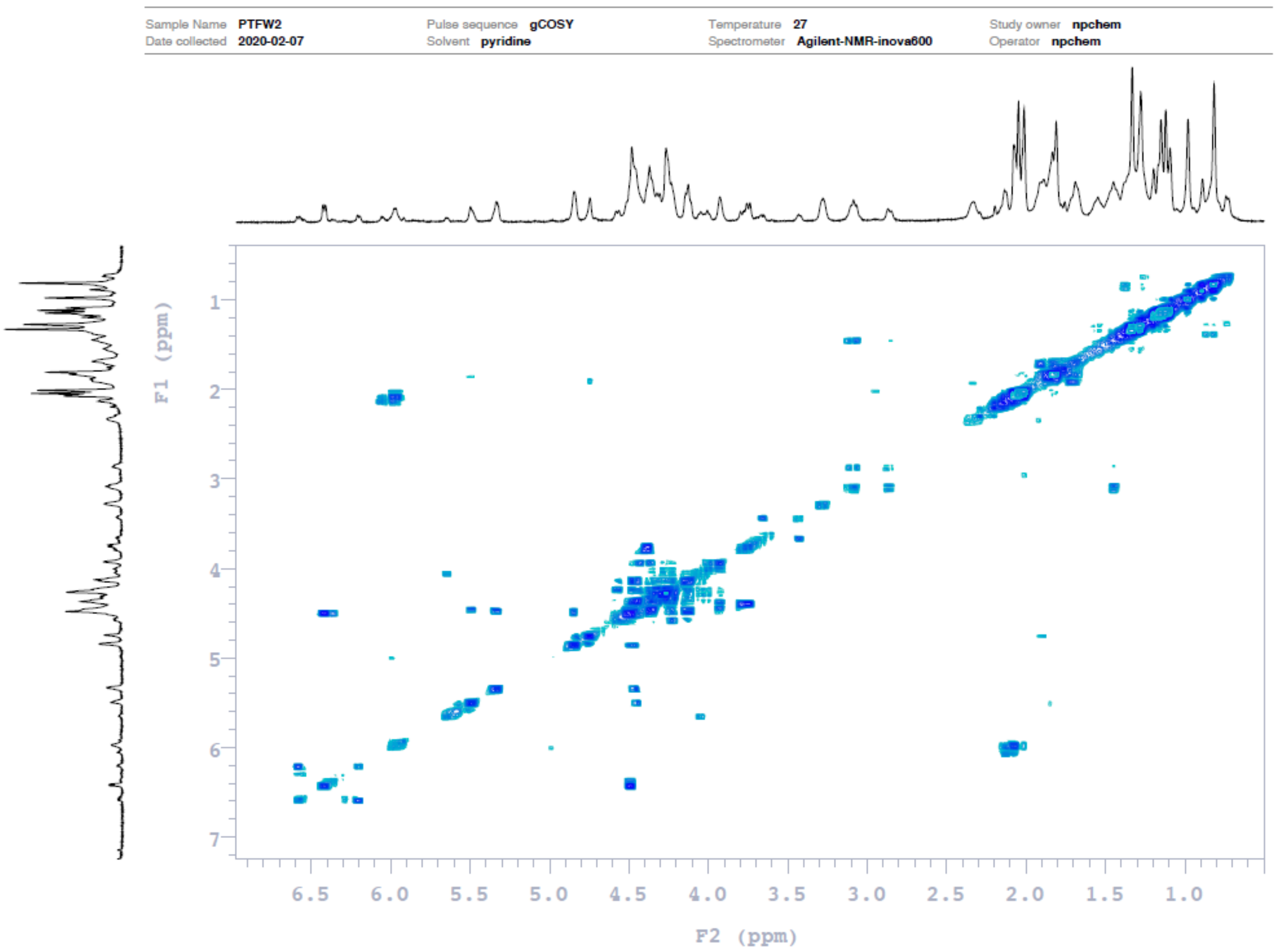

Figure S8. COSY spectrum of compound **2**

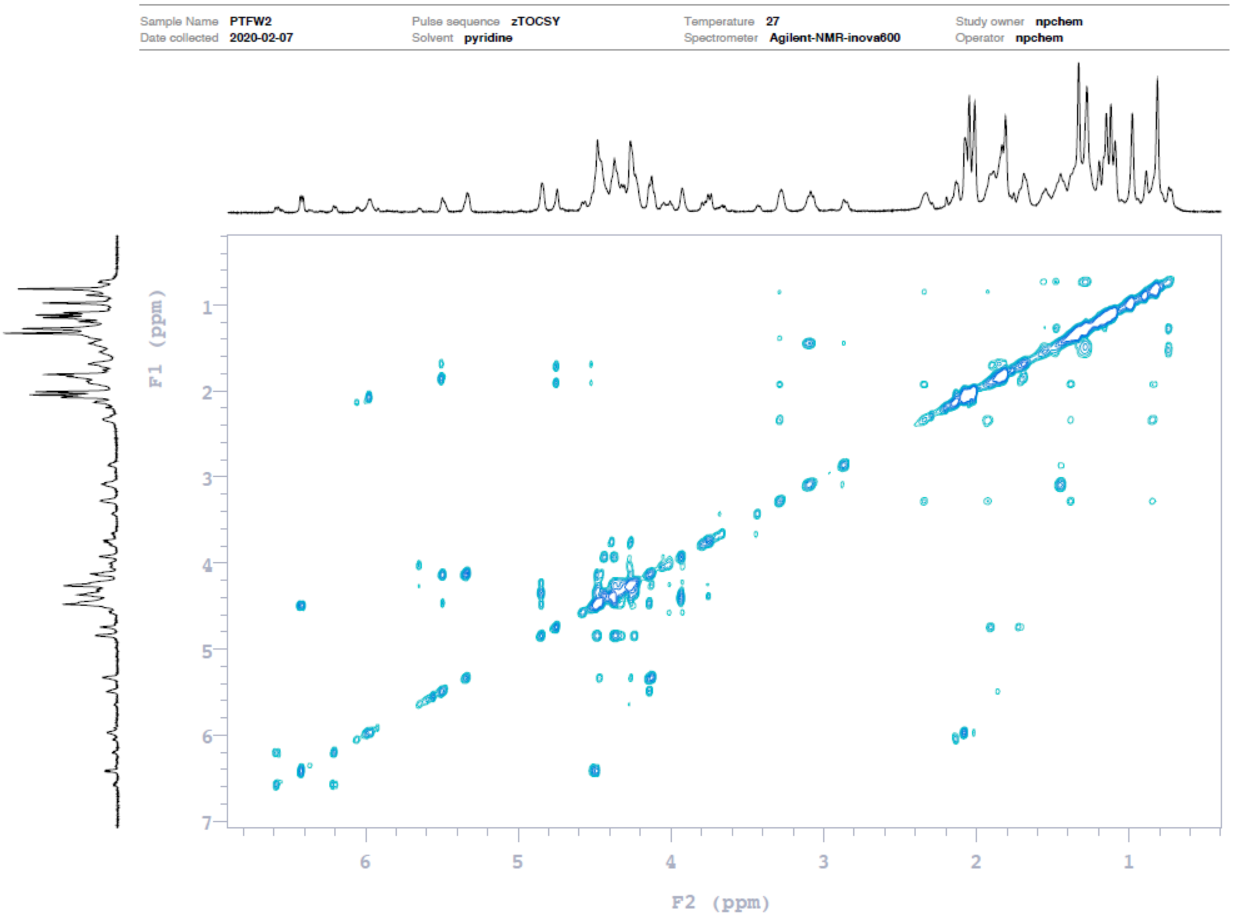

Figure S9. TOCSY spectrum of compound **2**

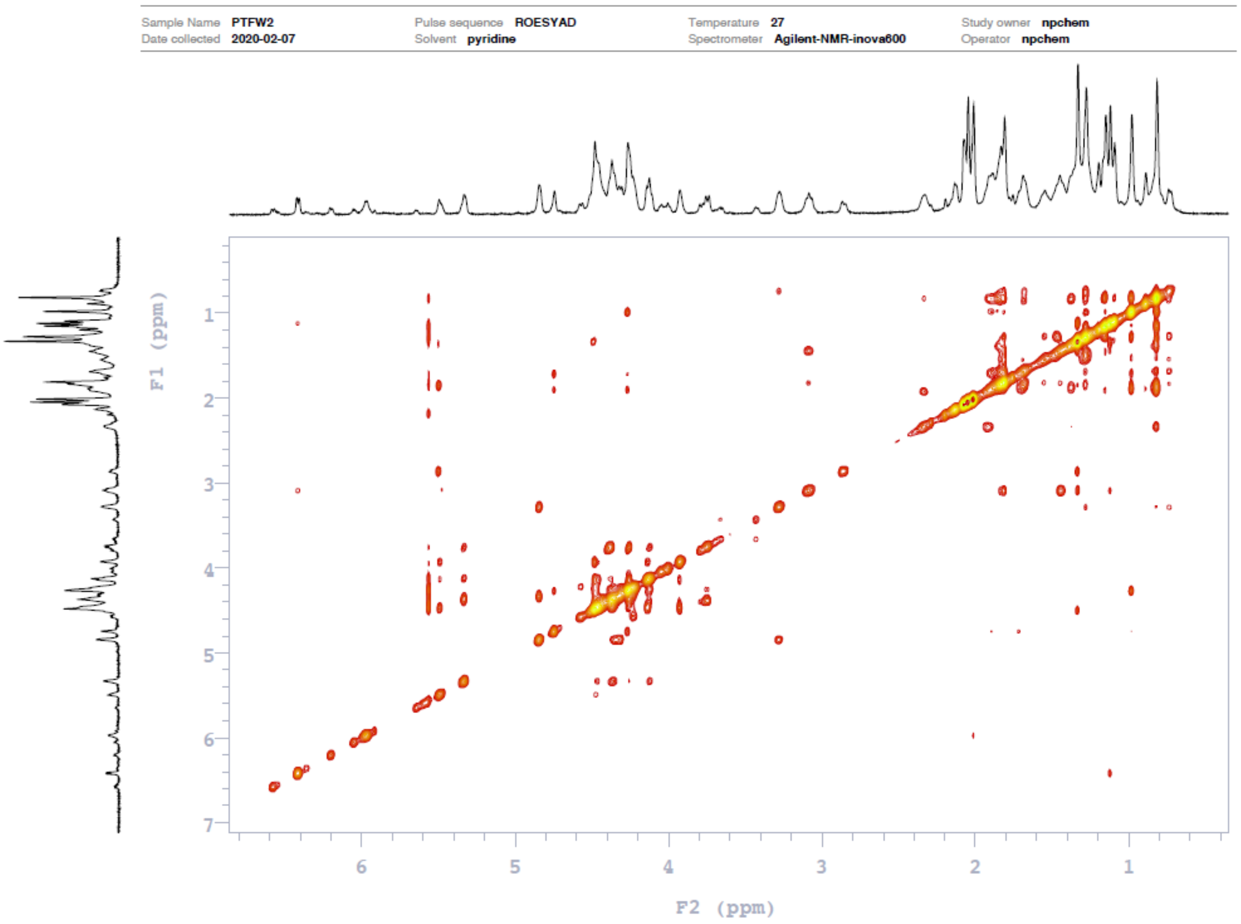

Figure S10. ROESY spectrum of compound 2

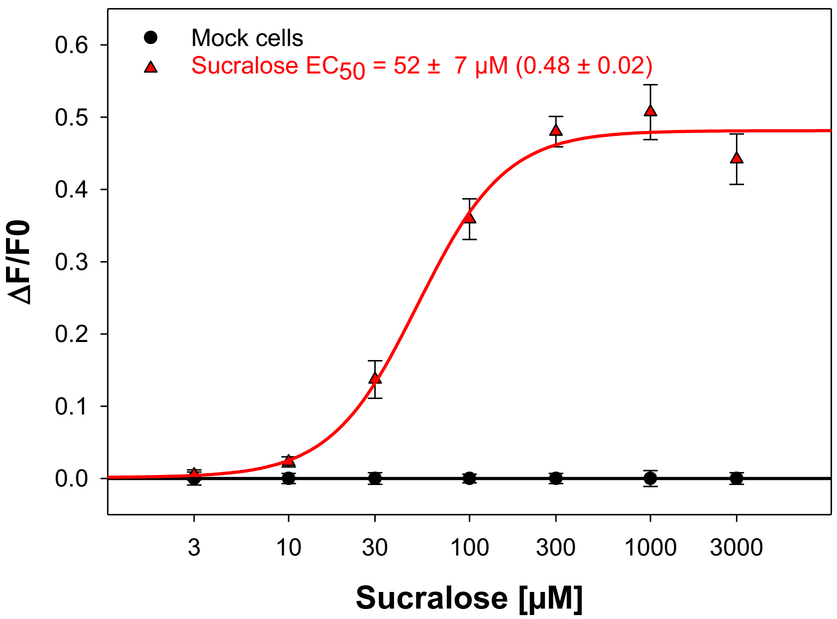

Figure S11. Activation of hTAS1R2-hTAS1R3 by sucralose

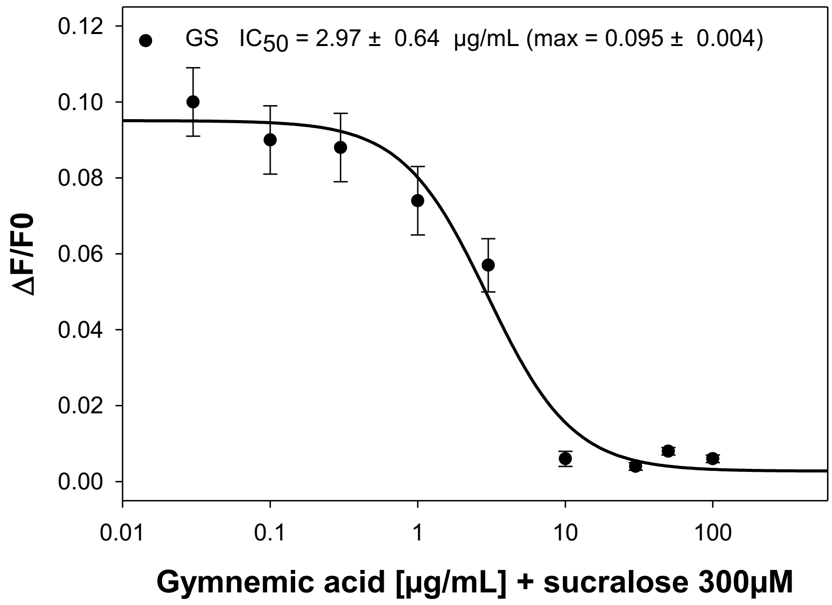

Figure S12. Inhibitory effect of GS on the response of sucralose by hTAS1R2-hTAS1R3
